# Complete Genome Sequence of Phocine Distemper Virus Isolated from a Harbor Seal (*Phoca vitulina*) during the 1988 North Sea Epidemic

**DOI:** 10.1128/genomeA.00291-13

**Published:** 2013-06-27

**Authors:** Rory D. de Vries, R. Joyce Verburgh, Marco W. G. van de Bildt, Albert D. M. E. Osterhaus, Rik L. de Swart

**Affiliations:** Department of Viroscience, Erasmus MC, Rotterdam, The Netherlandsa; Artemis, Research Institute for Wildlife Health in Europe, Utrecht, The Netherlandsb

## Abstract

Phocine distemper virus (PDV) was identified as the cause of a large morbillivirus outbreak among harbor seals in the North Sea in 1988. PDV is a member of the family *Paramyxoviridae*, genus *Morbillivirus*. Until now, no full-genome sequence of PDV has been available.

## GENOME ANNOUNCEMENT

Morbilliviruses are highly infectious, are spread via the respiratory route, and cause profound immunosuppression. Moreover, these viruses cause large outbreaks with high morbidity and mortality rates in previously unexposed populations. The prototype morbillivirus is measles virus (MV), an important pathogen of humans. Other members of the genus include canine distemper virus (CDV), rinderpest virus (RPV), dolphin morbillivirus (DMV), porpoise morbillivirus (PMV), peste des petits ruminants virus (PPRV), and feline morbillivirus (FmoPV) (1, 2). Phocine distemper virus (PDV) was identified as a member of the *Morbillivus* genus in 1988 (3, 4), infecting seals as a natural host and causing clinical signs and lesions similar to those of CDV in dogs and other carnivores (5). Since then, outbreaks and individual cases of PDV have been reported repeatedly (6–8). The morbillivirus genome consists of negative-sense single-stranded RNA, typically 15,500 to 16,000 nucleotides (nt) in length, comprising six genes that encode 8 proteins. Every 6-nt section of the viral genome is covered by one nucleocapsid (N) protein; therefore, the genome lengths of morbilliviruses always obey the “rule of six” (9).

We isolated PDV (previously referred to as PDV-1) from an organ suspension obtained from harbor seals that died during the 1988 North Sea outbreak (3). The organ suspension was previously used as challenge material in a PDV vaccination study (10); the isolated virus was passaged twice in Vero-dogSLAM cells (11). The RNA was isolated, and a sequence of the complete viral genome was obtained using 28 primer sets generating overlapping fragments, based on previously published partial PDV sequences (GenBank accession no. D10371, Y09630, and X75717). The 3′ and 5′ ends of the genome were sequenced following rapid amplification of cDNA ends (RACE). The genome was found to be 15,696 nt in length, consistent with the “rule of six,” and contained 6 nonoverlapping genes in the order N–P/V/C–M–F–H–L, which is typical for morbilliviruses. The amino acid lengths of the eight proteins encoded by the genome were N, 523 amino acids (aa); P, V, and C, 507 aa, 299 aa, and 174 aa, respectively; M, 335 aa; F, 631 aa; H, 607 aa; and L, 2,184 aa. Genes were flanked on either side by highly conserved transcription start and stop signals. It has been noted previously that the starting points of the six transcripts in morbilliviruses have a conserved phase (12). The phase of the PDV gene starts proved to be fully conserved compared to CDV. The genome contained a 55-nt leader region at the 3′ end, which was conserved when compared to other morbilliviruses (13) (84% homologous to CDV, 72% to MV). The trailer at the 5′ end was found to be 38 nt in length and was also highly conserved (14) (79% homologous to CDV, 82% to MV). This full-genome sequence can be used for comparative morbillivirus studies and may allow for the generation of an infectious molecular clone.

### Nucleotide sequence accession number. 

The complete genome sequence of the strain PDV/Wadden_Sea.NLD/1988 is available at GenBank under the accession no. KC802221.
